# Beyond Mor: Can Induction of Dopamine Homeostasis Along with Electrotherapy Attenuate the Opioid Crisis?

**Published:** 2023-03-02

**Authors:** Kenneth Blum, Abdalla Bowirrat, Eric R Braverman, Catherine Dennen, Foojan Zeine, Nicole Jafari, Keerthy Sunder, Panayotis K. Thanos, David Baron, Debmayla Barh, Ashim Gupta, Debasis Bagchi, Mark S Gold, Rajendra D. Badgaiyan

**Affiliations:** 1 Department of Molecular Biology and Adelson School of Medicine, Ariel University, Ariel, Israel; 2 Center for Sports, Exercise, Global Mental Health, Western University Health Sciences, Pomona, CA., USA; 3 The Kenneth Blum Behavioral & Neurogenetic Institute, LLC., Austin, TX, USA; 4 Institute of Psychology, ELTE Eötvös Loránd University, Budapest, Hungary; 5 Department of Psychiatry, University of Vermont School of Medicine, Burlington, VY., USA; 6 Department of Psychiatry, Wright University, Boonshoff School of Medicine, Dayton, OH., USA; 7 Division of Nutrigenomics, Victory Nutrition International, LLC., Bonita Springs, Fl., USA; 8 Centre for Genomics and Applied Gene Technology, Institute of Integrative Omics and Applied Biotechnology, Nonakuri, Purba Medinipur, West Bengal, In Department of Family Medicine, Jefferson Health Northeast, Philadelphia, PA, USA; 9 Awareness Integration Institute, San Clemente, CA, USA; 10 Department of Health Science, California State University at Long Beach, Long Beach, CA., USA; 11 Department of Human Development, California State University at Long Beach, Long Beach, CA., USA; 12 Division of Personalized Medicine, Cross-Cultural Research & Educational Institute, San Clemente, CA, USA; 13 Department of Psychiatry, University California, UC Riverside School of Medicine, Riverside, CA., USA; 14 Division of Neuromodulation Research, Karma Doctors & Karma TMS, Palm Springs, CA., USA; 15 Behavioral Neuropharmacology and Neuroimaging Laboratory on Addictions, Research Institute on Addictions, University at Buffalo, Buffalo, NY, Department of; 16 Genetics, Ecology and Evolution, Institute of Biological Sciences, Federal University of Minas Gerais, Belo Horizonte, Brazil.; 17 Future Biologics, Lawrenceville, GA USA; 18 Regenerative Orthopedics, Noida, UP, India; 19 Department of Pharmaceutical Sciences, College of Pharmacy & Health Sciences, Texas Southern University, Houston, TX.,USA; 20 Department of Psychiatry, Long School of Medicine, University of Texas Health Science Center, San Antonio, Texas, USA; 21 Department of Psychiatry, Washington University, School of Medicine, St. Louis, MO., USA; 22 Department of Psychiatry, Mt. Sinai University, Ichan School of Medicine, New York, NY., USA

**Keywords:** Opioid crisis, Genetic Addiction Risk Severity (GARS), Reward Deficiency Syndrome(RDS), H-Wave, NuCalm, KB220, Dopaminergic

## Abstract

One important area for consideration especially in terms of combating the ongoing never ending opioid crisis, relates to novel newer assessments for all addictive behaviors both substance and non-substance behaviors (RDS). It is very important to identify early in one’s life the possibility of, because of known DNA antecedents, the presence of pre-addiction. The development of the Genetic Addiction Risk Severity (GARS) test, Blum’s group believes that this type of testing should be the “standard of care” following additional studies. Understandably that while polymorphisms in the Mu-Opioid receptor (MOR) is of real concern in terms of setting people up for predisposition to opioid dependence, the genetic and epigenetic status of dopaminergic function must be considered as well. While this sounds bold (which it is) the results should be protected by the G.I. N. A. law enacted in the USA in 2011. One avenue of further investigation, instead of providing powerful opioids for opioid dependence, is to seek out non-addictive alternatives. Accordingly, other non-addictive modalities including genetic guided KB220 (amino-acid-enkephalinase-N-acetylcysteine-NAD), non-invasive rTMS for psychiatry and pain, epigenetic remodeling, gene edits, non-invasive H-wave for pain management and enhanced functionality, brain spotting, cognitive behavioral therapy awarenesss integration therapy, NUCALM, trauma therapy, awareness tools, genograms, exercise, sports, fitness programs (one hour per day), light therapy and even laughing therapy as well as any other known modalities that can induce reward symmetry. While the short term use of opioids for opioid dependence to reduce harm is certainly acceptable, clinicians should consider a better long-term plan.

## Introduction

One area for consideration relates to novel newer assessments for all addictive behaviors both substance and non-substance behaviors (RDS), that could distinguish between inflammatory pain relative to psychic pain [1]. It is indeed tantamount to our youth’s future mental health to develop a systematic validated coupling of neuropsychiatric measures with genetic addiction risk severity assessment specially to identify early on Preaddiction as suggested by Nora Volkow and George Koob directors of NIDA and NIAAA respectively [2]. In this regard, we believe that following more rigorous research, the Genetic Addiction Risk Severity (GARS) assessment may become a “standard of care” test even at birth [3]. Of course, it is to be understood that while polymorphisms in the Mu-Opioid Receptor (MOR) is of real concern in terms of setting people up for predisposition to opioid dependence, the genetic and epigenetic status of dopaminergic function must be considered as well. While this sounds bold (which it is) the results should be protected by the G.I. N. A. law enacted in the USA in 2011 [4]. Along with this type of genetic testing, this candidate approach is similar to the new FDA category of Genetic Health Risk (GHR) tests. One of us (ERB) suggested that in the future school systems should begin to adopt a novel Brain Health Check (BHC) utilizing validated neuropsychological assessments along with the RDSQ29 [5,6]. It is also important to be cognizant of epigenetic insults on reward genes such DRD2 methylation passed down to at least F2 generations. It is well-established that these histone modifications and other epigenetic modifications affect mRNA expression and in fact, it is known for example, that prenatal administration of THC can lead to increased sensitivity to morphine intake in animals as well vas deferens increased sensitivity to enkephalin & Norepinephrine activity [7,8].

Our future depends on finding non-addictive ways to modify or remove negative histone methylation or change mRNA transcription via gene editing [9]. With this stated we need “all hands on deck” to systematically develop a “unified theory of dopamine homeostasis” with the laudable goal of attenuating the long-term utilization of prescribing opioids for opioid dependence [10]. While it is true that during our worst opioid crisis since 1914, harm reduction in the short term is encouraged (6 months with tapering). Importantly, as reviewed herein, conversion to other non-addictive modalities including genetic guided KB220 (amin-acid-enkephalinase-N-acetylcysteine-NAD), non-invasive rTMS for psychiatry and pain, epigenetic remodeling, gene edits, non-invasive H-wave for pain management and enhanced functionality, brain spotting, cognitive behavioral therapy, NUCALM, trauma therapy, awareness tools, genograms, exercise, sports, fitness programs (one hour per day), light therapy and even laughing therapy as well as any other known modalities that can induce reward symmetry [11–15]. From our point of view certainly reducing prescribing powerful opioids including buprenorphine and methadone with increased utilization of non-addicting naltrexone coupled with pro-dopamine regulation (e. g. KB220z) should help free people from the chains of addiction and pain and once again redeem joy in the 2 billion worldwide people cursed with the shackles of RDS [16].

**Figure 1.**
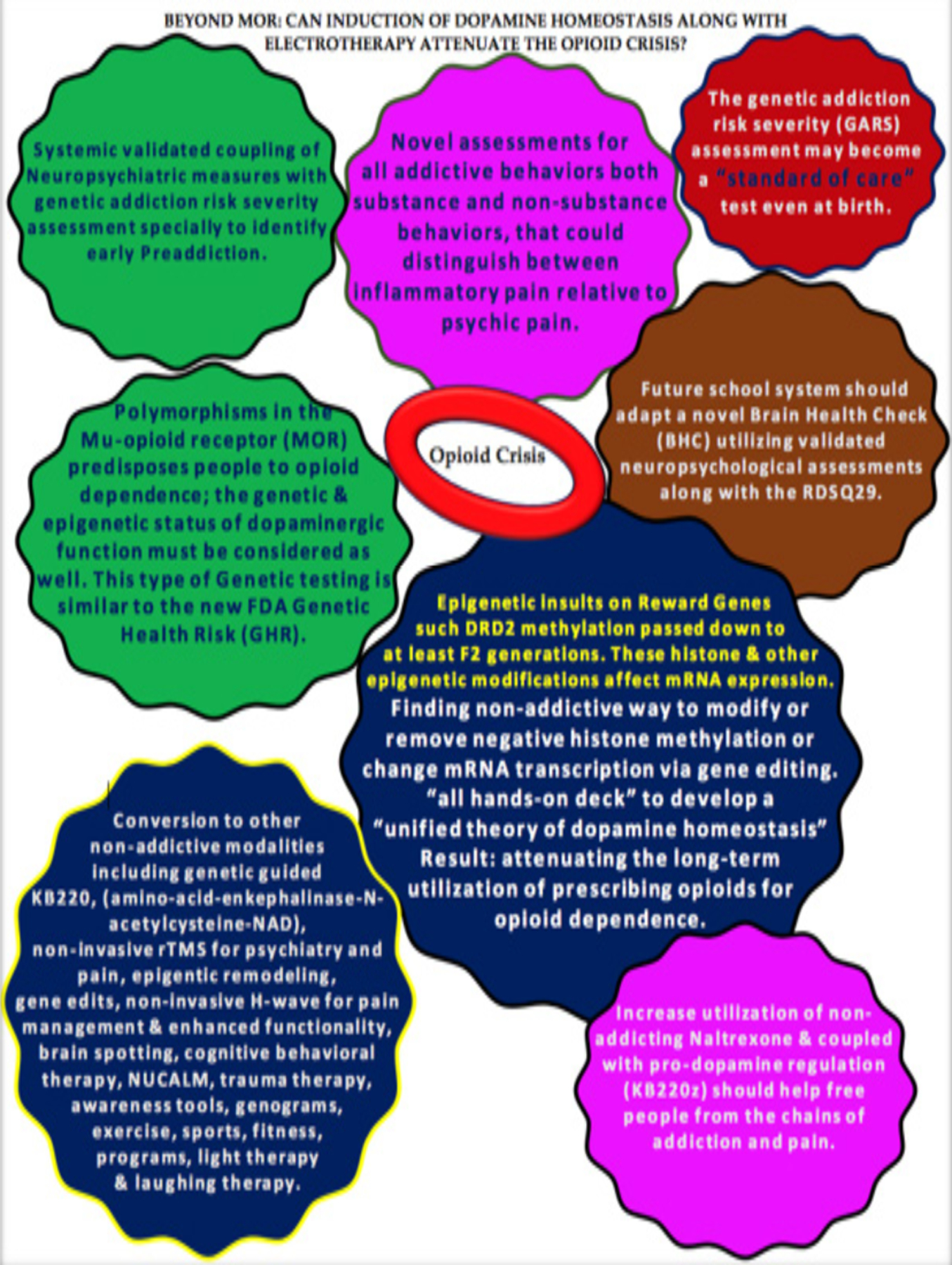
Beyond Mor: Can induction of dopamine homeostasis along with electrotherapy attenuate the opioid crisis?

Importantly, the Carter Center has determined that if the addiction crisis continues at the same rate, by 2030, it will cost America approximately 16 trillion dollars. The neurodevelopment of our children is compromised by mothers using opioids and other drugs during pregnancy [17]. A high rate of DNA polymorphic antecedents compounds epigenetic insults involving methylation onto specific essential genes related to normal brain function[18]. Myelination in the frontal cortex, known to take until the late twenties, delays proficient executive function and decision-making. Understanding this short-circuiting in brain development, along with potential high antecedent polymorphic risk variants or alleles and generational epigenetics, provides a clear rationale for embracing the Brain Research Commission (BRC) suggestion to mimic fitness programs with an adaptable Brain Health Check-up. Implementing the BHC within the educational systems in America and other countries might be a good starting point for proactive therapies to reduce juvenile mental health problems and eventually criminal activities, addiction, and other Reward Deficiency behaviors. To assist readers, we developed a schematic related to our futuristic model the reduce the unwanted and unnecessary abuse of powerfulopioids (Figure1).

A new legacy whereby the defected RNAs are “cured” by gene edits and homo-*sapiens* once again reach the promised land [19,20].
